# Results of Primary Extensor Tendon Repair of the Hand With Respect to the Zone of Injury

**DOI:** 10.5812/atr.7859

**Published:** 2012-10-14

**Authors:** Seyed Abdolhossein Mehdinasab, Mohammad Reza Pipelzadeh, Nasser Sarrafan

**Affiliations:** 1 Department of Orthopedic Surgery, Jundishapur University of Medical Sciences, Ahvaz, IR Iran; 2 Department of Anesthesiology, Jundishapur University of Medical Sciences, Ahvaz, IR Iran

**Keywords:** Extensor Tendon Injuries, Hand Injury, Lacerations

## Abstract

**Background:**

Laceration of the hand extensor tendons is common in the upper extremities, causing soft tissue trauma. These tendons, because of their superficial location and lying adjacent to bones, have a greater tendency to be injured than flexor tendons.

**Objectives:**

The aim of this study was to determine the results of primary repairs of lacerated extensor tendons of the fingers, with respect to the zone of injury, and also whether the results are different according to the anatomical zone in which they occur.

**Patients and Methods:**

During a period of two years and four months, 32 patients with open wounds and lacerated extensor tendons of the hand were hospitalized and underwent surgery. Repairs were done by a modified Kessler technique using 0 - 4 nylon suture. After repairing, the wrist was splinted for four weeks. Patients were followed-up for 12 months and the results were evaluated according to the Miller’s scoring system.

**Results:**

A total of 72 extensor tendons were repaired. The mean age of the patients was 24.6 years. The best results were obtained in zones 3 and 5 (84% and 88% respectively), and the worst results were seen in zones 1, 2 and 4, P = 0.01. Wound infections or re-ruptures were not seen.

**Conclusions:**

Repair of extensor tendon cuts on the dorsal surface of the hand and forearm were associated with better results in zones 3 and 5 than in zones 1, 2 and 4. Repair by the modified Kessler suture method provides proper stability at the site of the tendon cut.

## 1. Introduction

Extensor tendons of the hand, because of their superficial location, adjacency to bones, and less protection, have a greater tendency to injury than the flexor tendons. (1, 2). Although these tendons have easy surgical access, it is difficult to maintain their length and normal function (especially in the dorsum of the hand and fingers which have their own anatomic complexity, therefore, restoration of their length and gliding movements after repair is very important. Koul reported that single-stage repair of complex extensor tendon injuries can reduce morbidity and helps early functional outcomes (3). Miller’s scoring system to evaluate tendons (4) showed that; laceration severity, location of the injury, surgical technique, accompanying trauma, physiotherapy and the patient’s cooperation, were important factors in the outcome of these repairs. Of these factors, the location of the injury and post-operative immobilization are the two most important factors. Due to concomitant trauma such as; hand fracture, extensor tendon lacerations at the proximal interphalyngeal joint and proximal phalanx levels, these have the worst prognosis, and this was shown in Evans’ study (5). Saldana and Hung recommended dynamic braces and controlled motions to reduce the injury that follows operation immobilization, which had better results than static braces (6, 7). Newport and Purcell had good results from static splints after the operation (8, 9). The maximum power and minimum shortening of the tendons are two important goals in a repair. Cuts, open wounds from sharp objects such as; glass, knives, occupational insults, open fractures and blunt trauma, are the main causes of tendon injuries. Limitations in the active and passive movements of fingers, adhesion formation and finger deformities, which lead to dysfunction of pinch and grasp motions of hands, are mentioned as the most common complications of tendon lacerations (8, 10, 11).

## 2. Objectives

There are few studies concerning tendon repairs, which forced us to evaluate the primary repair of these tendons. 

## 3. Patients and Methods

This descriptive-prospective study was conducted in two referral university hospitals from 2004 till 2008. The study was approved by the Ethics Committee of Ahvaz Jundishapur University. Patients with open wounds and cuts in the dorsum of the hand or forearm, who had one or more lacerated tendons, were included in the study, while the exclusion criteria was; concomitant hand fracture. Thirty-eight patients were admitted and treatment was started. For all of the patients the probable complications were explained and informed consent was obtained. Patients were admitted by individual arrival or brought to our emergency ward by emergency medical services. No previous repair had been done for those patients. Six patients were excluded because they did not participate in follow-up. The study was composed of 32 patients, and a total of 72 tendons were repaired. Mean age of the patients was 26.7 years (14 - 46), SD = 9.2. Repairs were carried out in the operating room under general anesthesia in 29 patients and a double tourniquet in three patients using a modified Kessler suture by 0 - 4 and continuous running suture with 0 - 5 nylon. Nineteen of our patients were referred on the first day of injury, while 13 others came on the second to fourth days. Primary repairs were done for all patients. Following repairs in zones 3, 4, and 5, a volar splint was applied with the wrist at 40º and metacarpophalangeal joints in 15º of flexion. For immobilization in zones 1 and 2, the proximal inter-phalyngeal joint and distal interphalyngeal joints of the fingers were fixed by splints at extension. The patients were encouraged to move their fingers. The splint was removed at four weeks, then more daily activities were permitted, but a simple removable splint was applied during the night for two further weeks. Patients were referred for 30 visits of physiotherapy. Mean time of follow-up was 12 months (4 - 17). We used the Miller’s scoring system to evaluate the results which were divided into four groups; excellent, good, moderate and bad, according to decreased active extension (Lag) in the proximal interphalyngeal joint and metacarpophalyngeal joints of the hand and decreased flexion of these joints (Table 1). Descriptive statistics were employed and statistical data was presented in the form of frequency and percentages. Also, data were analyzed using SPSS software (IBM Corporation, USA) version 13 software, and a difference of 0.05 was considered as significant.

**Table 1:** Miller Classification for Extensor Tendon Injuries

	Total Extensor Lag (Degree)	Total Flexor Loss (Degree)
**Excellent**	0	0
**Good**	10 ≥	20 ≥
**Fair**	11 - 45	21 - 45
**Bad**	> 45	> 45

## 4. Results

In total, 32 patients (29 males and 3 females) with ages 17 to 46 years (mean age 26.4 years SD = 9.2) were treated and followed up. Right and left hands were involved in 18 and 14 patients, respectively. A total of 73 tendons were repaired. Zones 5 (36%) and 3 (34.7%) were the most common location of laceration, and zones 1 and 4 were the least common site of injury (Table 2). Complete laceration of the extensor retinaculum was seen in four patients, and some parts of it were repaired. According to the Miller scoring system, patients who had no limitations in their hand finger joints were classified as excellent. This class was seen in 53.8% of patients in zone 5 and 40% in zones 1 and 3. When active extension and flexion motions of fingers were at 45º< or more, they were classified as bad results. Lacerations in zone 4 (42.8%) and zone 2 (22.2%) had the worst results. Zone 5 (88.4%) and zone 3 (84%) had good and excellent results. We had moderate and bad results in rerupture of zones 1, 2 and 4 (P < 0.01). There were no infections or re-ruptures at the repair site.

**Table 2:** Results of Tendon Repair According the Miller Classification a

	No of Tendon, No. (%)	Excellent, No. (%)	Good, No. (%)	Fair, No. (%)	Bad, No. (%)
**Zone**					
**1**	5 (6.9)	2 (40)	0	1 (20)	2 (40)
**2**	9 (15.5)	3 (33.3)	2 (22.2)	2 (22.2)	2 (22.2)
**3**	25 (34.7)	10 (40)	11 (44)	3 (12)	1 (4)
**4**	7 (9.7)	1 (14.2)	2 (28.5)	1 (14.2)	3 (42.8)
**5**	26 (36)	14 (53.8)	9 (34.6)	2 (7.6)	1 (3.9)

^a^In zones 3 and 5 the rate of excellent and good results were greater than in zones 1, 2 and 4 P > 0.01

## 5. Discussion

It is believed that lacerations of hand extensor tendons have better results than flexor tendons. Lovett showed that ruptured tendons in the dorsum of the hand at zones 1, 2 and 3 can be treated by dynamic and static splints (12). In a recent study, under similar conditions, the results of the repair depended on the location or zone of the laceration. Dargan et al. showed that two thirds of extensor tendon primary repairs had good and excellent results (13). In another study by Newport et al. 110 extensor tendons were repaired and 52% had good to excellent results. They stated that acceptable results were greater in proximal zones 5 – 8 (according to the Werden scoring system) than in distal zones, while other zone results were similar (14). Hung et al. showed that lacerations in the dorsum of the fingers produced bad results (7). The study showed that better results occurred in zones 3 and 5, which is probably due to a lack of direct involvement of the hood extensor and requires a simpler repair. Tendon repair in the dorsum of the fingerscan lead to tendon shortening and adhesion formation, because it is a thin and superficial tendon, and has more adjacency to periosteum and skin. It is important to mention that gliding movements of the extensor tendons over the proximal phalanx is about 2-3 mm and its complete motion along its course is 50 mm, in comparison to deep flexor tendons, which have about 70 mm of gliding movement during flexion and extension of the finger. Consequently, extensor tendon repair is more vulnerable to creating scar tissue and shortening, than the flexor tendon, and this point may lead to limiting motion in the injured finger (5). In our study, the worse results in zones 1 and 2 were probably related to the complexity of the extensor anatomy and/or tendon shortening. We allowed active finger mobilization on the first day of surgery in the splint, and no tendon adhesion was seen, furthermore, no patient needed tenolysis. Good to excellent results in zone 4 were less than those in zone 5. Five patients had a complete laceration of the retinaculum and two patients had a partial laceration. As extensor tendons have a synovial lining just under the wrist retinaculum, trauma to this area and opening of the retinaculum for direct visualization and repair, can result in less gliding movement and a bowstring effect of the tendons. According to the time of post-operative immobilization, some authors suggest further support with a splint during the night for two weeks after splint removal. Nowadays, most authors recommend dynamic and controlled motion of the fingers from the first day of the operation, and they have shown good to excellent results with this method when tendon repairs have been carried out in zones 3 and higher (9). Although Watt and Purcell had good and acceptable results in all zones (in particular zone 1 and 2), they found that patients who were unable to fully comply can follow the static immobilization method after an operation (1, 15-17). We applied static volar splints after tendon repair and allowed early motion of the fingers as the patient could tolerate the pain. A modified Kessler method using 0 - 4 nylon was used reinforced with peripheral 0 - 5 monofilament running sutures. As there was no rupture of the tendon after repair, it seemed that this method is strong enough for repairing the extensor tendon.

In brief we found that:

1. We had more good and excellent tendon results in zones 3 and 5 (than in the other zones) which were similar to other studies, in particular with the Newport report.

2. Bad results in zones 1 and 2 may be due to less normal movement of the tendons at these sites or a shortening of the extensor tendons after repair, which leads to limitation of motion in the distal interphalyngeal joint and proximal interphalyngeal joints.

3. Repair of tendons under the wrist retinaculum can lead to a greater number of unsatisfactory results, because of injury in the extensor compartments or a bowstring effect of the repaired tendons.

4. The modified Kessler method, reinforced by peripheral running sutures, was strong enough to allow early postoperative motions of the fingers in the splint.
